# Analyzing and supporting mental representations and strategies in solving Bayesian problems

**DOI:** 10.3389/fpsyg.2023.1085470

**Published:** 2023-06-15

**Authors:** Julia Sirock, Markus Vogel, Tina Seufert

**Affiliations:** ^1^Mathematics Education, Institute of Mathematics and Computer Science, University of Education Heidelberg, Heidelberg, Germany; ^2^Institute for Learning and Instruction, Department for Psychology and Education, Ulm University, Ulm, Germany

**Keywords:** Bayesian problem-solving, visualization, mental model, cognitive load, coherence formation, multiple representations

## Abstract

Solving Bayesian problems poses many challenges, such as identifying relevant numerical information, classifying, and translating it into mathematical formula language, and forming a mental representation. This triggers research on how to support the solving of Bayesian problems. The facilitating effect of using numerical data in frequency format instead of probabilities is well documented, as is the facilitating effect of given visualizations of statistical data. The present study not only compares the visualizations of the 2 × 2 table and the unit square, but also focuses on the results obtained from the self-creation of these visualizations by the participants. Since it has not yet been investigated whether the better correspondence between external and internal visualization also has an effect on cognitive load when solving Bayesian tasks, passive and active cognitive load are additionally measured. Due to the analog character and the proportional representation of the numerical information by the unit square, it is assumed that the passive cognitive load is lower when using the unit square as visualization than when using the 2 × 2 table. The opposite is true for active cognitive load.

## 1. Introduction

Bayesian problems refer to situations of uncertainty where inferential judgment is needed. The belonging Bayes Theorem describes the probability of an event, based on prior knowledge of conditions that might be related to the event being of interest. One of the most popular examples is the Mammographie problem (Gigerenzer and Hoffrage, 1995, p. 685; adapted from Eddy, 1982):

The probability of breast cancer is 1 % for a woman at age forty who participates in routine screening. If a woman has breast cancer, the probability is 80% that she will get a positive mammography. If a woman does not have breast cancer, the probability is 9.6% that she will also get a positive mammography. A woman in this age group had a positive mammography in a routine screening. What is the probability that she actually has breast cancer? ____%

In order to calculate the risk of having cancer for this woman, the Bayes Theorem is required. The probability of having cancer with a positive test result $P\left(C|T+\right)$ is:

$$\begin{array}{c} P\left(C|T+\right)=\frac{P\left(T+|C\right)\;\cdot \;P\left(C\right)}{P\left(T+|C\right)\;\cdot \;P\left(C\right)+P\left(T+|\neg C\right)\;\cdot \;P\left(\neg C\right)}\\ =\frac{0,8\;\cdot \;0,01}{0,8\;\cdot \;0,01+0,096\;\cdot \;0,99}=0,078=7,8\% \end{array}$$

The result is counterintuitively low (Eddy, 1982) and research shows a miscalculation of probabilities as well as a lack of understanding the results (McDowell and Jacobs, 2017). Without any further help people only guess the correct answer or try to combine the given numbers in the text without a deeper understanding of the problem.

In this paper, we want to address the cognitive processes, which are necessary when solving Bayesian problems. Thus, based on theoretical models and empirical findings we first analyze these processes and suggest different instructional approaches to foster them by using multiple representations. The goal of our study is first to substantiate whether these processes are actually crucial while solving Bayesian problems and second to examine the effects of the supporting approaches with regard to the correct solutions of the problems and to the experienced cognitive load.

### 1.1. Bayesian problems require to translate between the mathematical-model world and the real-model world

Central to Bayesian tasks is the translation process from problem statements in the real world into the formula language of the mathematical world, which fits the main aspects of modeling in school (*cf.* Eichler and Vogel, 2015).

Both, the process of identifying the relevant numerical information and the translation into the formula language is particularly difficult, when people cannot grasp the meaning of the numerics, because they are too abstract. One approach to overcome this difficulty is to substitute probabilities in the text with natural frequencies as demonstrated by e.g., Brase (2021), Johnson and Tubau (2015), and Gigerenzer and Hoffrage (1995). The Mammographie problem in terms of natural frequencies looks like the following:

100 out of 10,000 women who participate in routine screening have breast cancer. Out of 100 women who participate in routine screening and have breast cancer, 80 will have a positive result. Out of 9,900 women who participate in routine screening and have no breast cancer, 950 will also have a positive result. How many of the women who participate in routine screening and receive a positive test result have breast cancer? Answer: ____ out of ____

The substitution of probabilities by natural frequencies already represents a translation of the mathematical world into the real world. While probabilities induce a computation, natural frequencies can be observed directly. Via natural frequencies all numerical information are absolutely quantified to a single reference class (i.e., the superordinate set of 100 persons), where categories are naturally classified into expected values of the compounded events $C\cap T+,C\cap T-,\bar{C}\cap T+,\bar{C}\cap T-$ . In this case, the conditional distribution does not depend on the between-group (having cancer, not having cancer) base rates, but only on the within-group frequencies (true-positive-rate, false-positive rate). Accordingly, the base rates can be ignored, and the required computations are reduced to a simpler form of Bayes rule (*cf.* Johnson and Tubau, 2015). The solution via calculation with natural frequencies would be:

$$P\left(C|T+\right)=\frac{80}{80+950}=7,8\%$$

### 1.2. Bayesian problems use conditioned probabilities

The second challenge when solving Bayesian problems is due to its logical structure and to identify the crucial elements that constitute the problem. The problem structure is one of so-called nested sets, which represent one explanation for the facilitating effect of natural frequencies (Sloman et al., 2003, p. 297). For the above-mentioned example, there are four subsets of events, defined by the two subsets of each main criteria, i.e., being ill (yes/no) and having a positive test result (yes/no). From a representational perspective, one has to construct a relational mental framework of units that are on a higher level and which can be subdivided into sub-ordinate units. The resulting mental representation thus has a spatial structure. In terms of current cognitive models of knowledge acquisition (e.g., Schnotz and Bannert, 2003) it would be called a depictive mental representation as it is analog to the nested structure of the problem.

According to multimedia principle of Mayer (2014), it could be helpful to provide a depictive representation, i.e., a visualization in addition to the textual problem statement, if learners need to construct a depictive mental representation. However, it is not sufficient to provide any kind of visualization, it should map to the structure of the mental representation (Gentner, 1983). Different studies indicate that visualizations could actually help solving Bayesian problems (Brase, 2008; Khan et al., 2015; Eichler et al., 2020; Brase, 2021). Eichler et al. (2020) showed that particularly visualizations that illustrate the nested-sets structure of a Bayesian situation facilitate Bayesian Reasoning.

**Figure 1:**
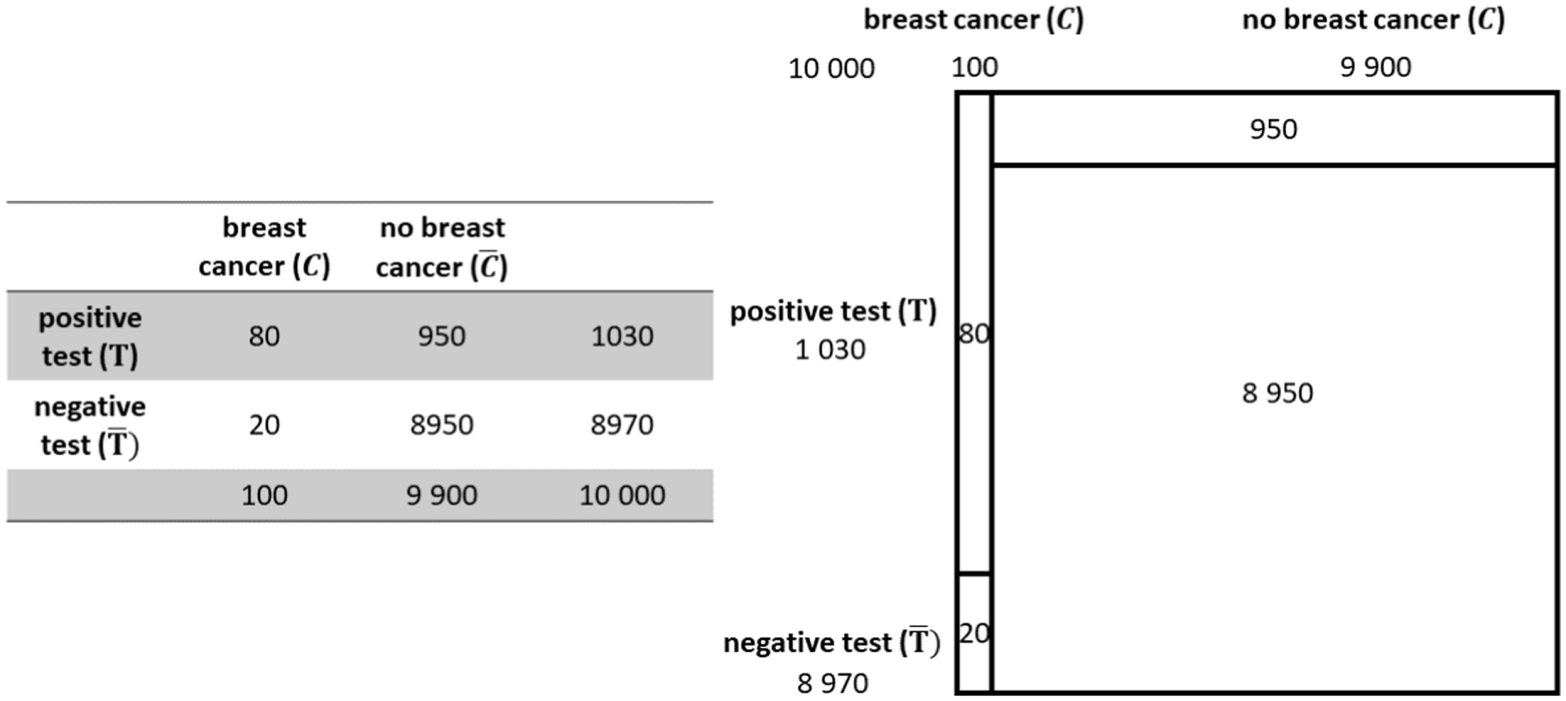
2 × 2 table (left) and unit square (right) belonging to the mammographie problem.

With the visualization by a 2 × 2 table for example, the given information is ordered and thus already pre-structured in columns and rows in such a way that the reader can see at a glance, for example, the positive test rates (Figure 1). It already contains all values relating to the base rate, number of positive and negative test results, and number of diseases and no diseases but not values such as the false-positive rate. Therefore, it is still up to the reader to bring the elements into relations or to overview covariations.

The unit square is a statistical graph (Tufte, 2015), which means, that the sizes of the partitioned areas are proportional to the sizes of the represented data. Therefore, the proportions of incidences, like, e.g., the base-rate, in a population are represented numerically as well as geometrically (Figure 1). Thus, the numerical descriptively represented information gets an analogs depictive counterpart which according to the supplantation effect (Vogel et al., 2007) may lead to a deeper elaboration of the Bayesian situation’s mental model via mutual supplementation (*cf.* above; Schnotz and Bannert, 1999).

Both visualizations turned out to be effective for solving Bayesian problems in terms of an increase in performance (Binder et al., 2015; Eichler et al., 2020). It would also be plausible that it should also be easier to construct the required knowledge representation when provided by an analog representation. However, up to now there is no analysis whether the better match of external and internal representation also affects cognitive load. Taking into account the different aspects of cognitive load (Paas and Van Merriënboer, 1994) one could assume that the visualizations unburden learners from searching for the relevant information and their inter-relation and should thus reduce extraneous load. It might also be possible that the visual ordering and clustering of information within the graph helps to reduce the perceived complexity and thus intrinsic load. Both types of load, extraneous and intrinsic are due to task affordances which learners experience passively. Thus, in a recent paper by Klepsch and Seufert (2021), the required resources are referred to as passive load. In contrast, learners can also decide to invest effort, i.e., they devote resources actively to deal with the task. Thus, these resources are referred to as active load. With regard to the visualizations of Bayesian problems, learners might be activated to use them and consequently, the active load might be increased.

### 1.3. Bayesian problems require to relate different probabilities

One additional requirement when solving Bayesian problems is to apply the Bayes formula. People need to understand what elements determine the denominator and the numerator of the Bayes ratio and what the underlying meaning of this ratio is. The required mental model does not only include single separated elements but their interplay (Schnotz and Bannert, 2003). A mental model is also characterized by its flexibility. Learners can “see” the problem structure and manipulate it mentally (“envisioning” and “running”; de Kleer and Brown, 1983, p. 156). With regard to solving Bayesian problems learners could thus be able to specify how the result would change with varying parameters of the problem.

One essential issue of the unit square is that the numerically represented products of conditioning probabilities and conditioned probabilities [e.g., 𝑃(T + |C) · 𝑃(C)] which determine the denominator and the numerator of the Bayes formula correspond to the calculation of the rectangular subareas (length multiplied by width) of the unit square. With this kind of calculation, the students are usually very familiar when learning about conditional probabilities and Bayesian situations. Thus, the in the novices’ eyes complex looking Bayesian formula gets potentially better accessible for students because its parts are based on well-known mathematical subroutines. Therefore, the benefit in a mathematical regard is that the unit square can be used to calculate the numerical value of probabilities and to determine the Bayes ratio (*cf.* Oldford, 2003, p. 1).

### 1.4. Bayesian problems require to deal with multiple representations

Beside the supportive effects of additional visualizations, they also need to be processed. From a multi-representational point of view, like in this case the textual problem statement, the visualization and the formula, dealing with it requires to mentally link the individual representations and build a coherent mental representation; a process called coherence formation (Seufert, 2003).

With regard to the promotion of coherence formation processes, there is a variety of empirical work (see Seufert and Brünken, 2006 for a summary). They show that different approaches are possible to successfully support knowledge acquisition with multiple representations. On the one hand, the improved and deepened processing of individual representations (local coherence formation) is promoted and on the other hand, the actual linking of multiple representations (global coherence formation). Empirical studies show positive effects of prompting learners to find corresponding elements or relations (Bodemer and Faust, 2006; Schnaubert and Bodemer, 2017). However, to support learners in finding these corresponding elements and relations, it also turned out to be effective to highlight the correspondences by, e.g., color coding (Vogt et al., 2020) or by explicitly explaining relations between representations on a deeper level of understanding (Seufert, 2003). Particularly, the combination of highlighting and deep-level help turned out to be supportive in terms of increased learning outcomes and decreased overall cognitive load (Seufert and Brünken, 2006).

## 2. Present study

### 2.1. Hypothesis

The focus of this study is on the comparison of information-equivalent visualizations to support the solution of Bayesian problems, which differ qualitatively with respect to analog and non-analog representation formats. Specifically, the Bayesian formula, the 2 × 2 table and the unit square are examined. The formula itself does not provide any visualized help. The 2 × 2 table, on the other hand, presents the important information from the text in a spatially structured way. Studies already show that the 2 × 2 table supports performance (Binder et al., 2015; Eichler et al., 2020). Based on the additional analogous character of the unit square, the second hypothesis would lead to the assumption that the unit square outperforms the 2 × 2 table. However, Böcherer-Linder and Eichler (2019) found the unexpected effect that it was the other way round. In their discussion, they assume that this unexpected result was partially influenced by the context of a certain item with the most extreme distribution as well as by the unfamiliarity of the unit square, and they argued for further investigations. Using other items in another setting of data collection (within-subject design) including a training how to construct a visualization (*cf.* below section 2) we argue for the unit square on the theoretical considerations and state:

*Hypothesis 1:* Performance will be the highest in tasks that show the unit square for support, followed by tasks that use the 2 × 2 table while in tasks that use the formula the performance is lower.

Beyond performance, the cognitive load associated with the different visualizations is investigated. Since the 2 × 2 table already has a spatial structure and thus a better correspondence between the external and internal representation, it is assumed that the passive cognitive load is lower compared to the Bayesian formula. Consistent with this, because of the additional analogous nature of the unit square, it is reasonable to assume that passive cognitive load is even lower.

*Hypothesis 2:* In tasks that show the unit square for support, passive cognitive load will be lower than in tasks that use the 2 × 2 table and tasks that use the Bayesian formula have the highest passive cognitive load.

With regard to active cognitive load, it is the other way around—the lower the passive cognitive load, the more focus can be placed on active cognitive load. This leads to the hypothesis:

*Hypothesis 3:* In tasks that use the unit square for support, active cognitive load is higher than in tasks that use the 2 × 2 table. The active cognitive load is higher with the 2 × 2 table than with tasks that use Bayes’ formula.

It is also expected that learners’ prior knowledge as well as their abilities in spatial and logical reasoning affect the relation between the different visualizations and performance or passive and active load. We therefore assume moderating effects of all these aptitudes: With lower prior knowledge or spatial and logical abilities the differences between the visualizations will be larger than with increasing knowledge and abilities.

*Hypothesis 4 (a-c):* Learners’ prior knowledge will moderate the relation between the different visualizations and their performance (4a), their passive load (4b) and their active load (4c).

*Hypothesis 5 (a-c):* Learners’ spatial abilities will moderate the relation between the different visualizations and their performance (5a), their passive load (5b) and their active load (5c).

*Hypothesis 6 (a-c):* Learners’ logical abilities will moderate the relation between the different visualizations and their performance (6a), their passive load (6b) and their active load (6c).

In addition to the analysis of the quantitative measures, a qualitative-exploratory analysis of the think aloud recordings will be made. This is intended to validate the problem areas as well as to analyze indications of the extent to which the different visualizations support the problem.

### 2.2. Method and analysis

Participants will be first semester psychology and mathematics teacher education students. A total of 66 subjects are to be surveyed in a within-subject design. The number of participants has been determined with G*Power, assuming a small effect size of 0.2 (based on comparison analyses of different visualizations from by Eichler et al., 2020) in an ANOVA with one group and three repeated measures. The survey will take place via videoconference, with both a whiteboard for drawing and voice recordings available to capture participants’ approach using think aloud methods.

The study consists of a total of seven typical tasks to capture Bayesian thinking. The first task is presented without any assistance to see how the students already deal with the task in terms of prior knowledge. Once students have created their solution to the task, they are provided with step-by-step prompts to develop supporting visualizations. First, the students create a 2 × 2 table by using the prompts, which is then developed into a unit square. At this point, the visualizations are created by the students themselves, allowing for deeper understanding (Bodemer et al., 2005).

In a short video, the individual steps are now presented in summary form, with corresponding parts of the 2 × 2 table, the unit square and Bayes’ formula being highlighted via appropriate color coding. In the video, first the transition of the text task to the representation by a 2 × 2 table is described, then the transition from the 2 × 2 table to the unit square and finally to Bayes’ mathematical formula.

After the video, participants are given six Bayesian tasks with comparable difficulty but different contexts and numbers (two tasks each in similar contexts) and different visualization support (2 × 2 table, unit square, and Bayes formula). Randomization of contexts is intended to eliminate bias in the results. Table 1 shows the procedure.

**Table 1:** Summary of the procedure of the study.

Bayesian Task 0 (prior knowledge measure) No visualization Context 1			
Prompts			
Summarizing video			
Bayesian Task (visualization)	Groups with different contexts per task for randomized order control		
1 (2 × 2 table)	Context 2	Context 4	Context 6
2 (2× 2 table)	Context 3	Context 5	Context 7
3 (unit square)	Context 4	Context 6	Context 2
4 (unit square)	Context 5	Context 7	Context 3
5 (Bayes‘formula)	Context 6	Context 2	Context 4
6 (Bayes‘formula)	Context 7	Context 3	Context 5
	Control variables (course of study, Abitur grade in maths, and test for spatial and logical thinking)		

Following each task, active and passive cognitive load as the dependent variables are measured by the two items “I exerted myself” (active cognitive load) and “It was exhausting” (passive cognitive load; *cf.* Klepsch and Seufert, 2021).

Performance as another dependent variable is determined by the solution quality of the six Bayesian tasks. For this purpose, one point each is awarded for the subsample of the numerator and the denominator of the fraction of the Bayes’ formula to be determined and one point for the correct relation. Performance for the first, unassisted task is calculated in the same way and is used as prior knowledge measure. Participants will be excluded when there is less than one measure per visualization for each, performance and load-measure.

As additional control variables, the course of study and Abitur grade in math will be inquired about. The course of study is surveyed for descriptive purposes only. The graduation grade in mathematics is checked for correlation with performance in Bayesian tasks. If there is a correlation, the influence of the grade in relation to the performance is controlled as a covariate. Furthermore, learners’ spatial and logical thinking abilities will be analyzed by using the KFT-cognitive abilities subtests for paper folding and numerical series by Heller and Perleth (2000). The ability score is the percent of correctly solved tasks in these tests. The students’ prior knowledge is already assessed via the first task. Missings for control variables will be imputed, despite the respective moderation analyses. The moderation analyses with the moderators prior knowledge, spatial and logical thinking abilities are conducted exploratively.

All participants are given the same tasks so that the analyses can all be done within subject by repeated measures ANOVAs with subsequent contrast analyses. To determine the sphericity, a Mauchly test is first performed. If the significance of the sphericity is below 0.05, the Huynh-Feldt correction is used for a sphericity of *ε* > 0.75, and the Greenhouse–Geisser correction is used for a value of *ε* < 0.75. If we obtain a result significant to the alpha error level *p* < 0.10, more detailed correlations are calculated using contrast analyses with the aid of the Bonferroni correction. The control variables prior knowledge, spatial and logical thinking are analyzed as moderators in individual moderation analyses via the PROCESS plug-in of Hayes (2022) in SPSS. For each moderation analysis the outcome variable Y is the performance in the Bayesian tasks, the independent variable X is the different visualizations, and the M variables are prior knowledge, spatial and logical thinking, respectively. When there is a significant correlation between one of these potential moderators with the dependent measure the respective variable will be additionally included in the moderation analyses as a covariate (only in those where the variable is not analyzed as a moderator).

We set a significance level of *p* = 0.05 for all analyses. The contrasts will be tested one-sided as the hypotheses are directed. All non-significant results will lead to rejection of the respective hypothesis. Effect sizes will be calculated for all analyses. If effects are medium size but non-significant, the hypotheses will be interpreted as worth considering in future studies under additional specifying conditions like learner characteristics, more or less difficult tasks etc., depending of the overall results.

The qualitative data will be aggregated in an iterative process with regard to the problems defined in the theoretical analysis on the one hand and the think aloud data revealed on the other hand. In addition, possible statements about the usability of the different visualizations will be aggregated with regard to their theoretical and reported functionality.

## Data availability statement

The original contributions presented in the study are included in the article/supplementary material; further inquiries can be directed to the corresponding author.

## Ethics statement

Ethical review and approval was not required for the study on human participants in accordance with the local legislation and institutional requirements. The patients/participants provided their written informed consent to participate in this study.

## Author contributions

JS, MV, and TS planned and designed the study. MV and TS developed the theoretical framework and critically reviewed the work. JS drafted the manuscript. All authors contributed to the article and approved the submitted version.

## Conflict of interest

The authors declare that the research was conducted in the absence of any commercial or financial relationships that could be construed as a potential conflict of interest.

## Publisher’s note

All claims expressed in this article are solely those of the authors and do not necessarily represent those of their affiliated organizations, or those of the publisher, the editors and the reviewers. Any product that may be evaluated in this article, or claim that may be made by its manufacturer, is not guaranteed or endorsed by the publisher.
